# Comparative outcomes of hip arthroscopy for femoroacetabular impingement in football and non-football athletes: a clinical analysis

**DOI:** 10.1007/s00402-025-05866-0

**Published:** 2025-04-25

**Authors:** Giancarlo Riccobono, Alfred Ferré-Anoirte, Roberto Seijas, David Barastegui, Ramon Cugat

**Affiliations:** 1 Northwestern University Feinberg School of Medicine, Chicago, USA; 2 Fundación García Cugat, Barcelona, Spain; 3 Instituto Cugat, Hospital Quirónsalud, Barcelona, Spain; 4 Mutualidad de Futbolistas Españoles, Delegación Catalana, Barcelona, Spain

**Keywords:** Femoroacetabular impingement, FAI, Hip arthroscopy

## Abstract

**Introduction:**

Femoroacetabular impingement (FAI) is a common cause of hip pain and dysfunction, particularly among athletes, including football players. The condition is characterized by abnormal contact between the femoral head-neck junction and the acetabulum, leading to cartilage damage and labral tears. Hip arthroscopy has emerged as a minimally invasive treatment option, offering faster recovery and improved outcomes compared to traditional surgery. This study aims to compare outcomes between football players and non-football athletes undergoing hip arthroscopy for FAI.

**Materials and methods:**

This retrospective, single-center study analyzed a database of patients undergoing hip arthroscopy for FAI between 2007 and 2023. The study compared football players (n = 16) and non-football athletes (n = 16), matched for age, sex, and BMI. Pre-operative assessment included the Hip Outcome Score (HOS), Visual Analog Scale (VAS), and other functional questionnaires. Radiographic evaluations included the alpha and Wiberg angles, and intra-operative findings were recorded. The surgical approach involved femoral and acetabular osteoplasty, labral repair, or labrectomy, depending on injury morphology.

**Results:**

Both groups showed similar pre-operative pain levels (VAS) and functional scores. However, significant differences were observed in the Tegner and Hip Sports Activity Scores (HSAS), with football players showing higher activity levels pre-operatively. Both groups demonstrated significant improvements in alpha and Wiberg angles post-surgery (p < 0.001). The surgery duration was similar between groups, and no significant differences in post-operative outcomes were found between football and non-football players.

**Conclusions:**

Hip arthroscopy is effective for both football and non-football players with FAI, with both groups experiencing significant improvements in hip joint function and pain relief. While pre-operative functional scores differed, particularly in activity levels, both groups benefited from similar post-operative outcomes, suggesting that the surgical approach is suitable for active individuals across different sports. Further research is needed to explore long-term outcomes and return-to-sport rates in these populations.

## Introduction

Femoroacetabular impingement (FAI) is increasingly recognized as a significant cause of hip pain and dysfunction in individuals across various age groups [1]. The condition involves abnormal contact between the femoral head-neck junction and the acetabulum, resulting in mechanical damage to the hip joint's articular cartilage and labrum [2]. This repetitive impingement can lead to decreased range of motion and locking of the hip joint. Additionally, it can cause labral tears, cartilage damage, and, if left untreated, may progress to degenerative hip joint disease [3, 4].

FAI is broadly classified into two types: cam and pincer impingement. Both types of impingement can cause pain, decreased range of motion, and, ultimately, hip joint damage [5]. In the past, the treatment options for FAI were limited to conservative options, often resulting in prolonged periods of pain and decreased athletic performance [6]. However, the advent of hip arthroscopy has revolutionized the management of this condition, particularly in football (soccer) players [2, 7].

During the hip arthroscopy procedure, the surgeon can reshape the femoral head, remove excess bone, repair damaged cartilage, and correct any other anatomical abnormalities contributing to impingement [8, 9]. Hip arthroscopy and its minimally invasive nature, as opposed to open surgery or the best conservative therapy, results in reduced tissue trauma, faster recovery, and earlier return to sports for football players [10–15]. It has become the preferred surgical intervention for many athletes and non-athletes who wish to resume their lifestyle as quickly as possible [16]. High return to sport rates have been described after this surgical procedure (74.7%) in football players, with a mean time to return to play of 33.1 ± 26.3 weeks [16].

Football is a physically demanding sport that places significant stress on the hip joint [17]. The repetitive kicking, twisting, and rapid changes in direction often experienced by football players can lead to excessive friction and abnormal movement within the hip joint[18]. Over time, this can result in the development of FAI, making it imperative to understand the specific considerations and outcomes associated with hip arthroscopy in this athletic population. The number of studies comparing football players to non-athletes after hip arthroscopy is lacking [19]. A myriad of studies have examined return to sport in football players after undergoing hip arthroscopy for FAI; however, few have examined the differences in presentations across football players in comparison to non-football players.

Describing the differences in injury characteristics and outcomes between football players and non-football players who undergo hip arthroscopy due to FAI may provide valuable insights into the sports-related demands on the FAI treatment approach and long-term prognosis [20, 21]. Thus, the aim of this study is to compare pre-operative and intra-operative scores in football players to those in non-football players as well as the changes in the alpha and Wiberg angles from pre-surgery to post-surgery. This analysis can help improve the standard care of treatment and provide orthopedic surgeons an additional tool to make data-driven decisions regarding the risks and benefits of such a surgery across two actively-different populations. We hypothesize that football players undergoing hip arthroscopy for FAI will present with distinct pre-operative and intra-operative characteristics, including greater alpha and Wiberg angle abnormalities, compared to non-football players, and will demonstrate greater post-operative improvements in these radiographic measures, reflecting the influence of sport-specific demands on FAI pathology and outcomes.

## Methods

### Study design

This study was designed as a retrospective, single-centre comparative analysis of a prospective database.

### Participants

Between 2007 and 2023, all patients undergoing hip arthroscopy for FAI at our facilities were approached for eligibility. Football players were included if they had a FAI diagnosis (clinical symptoms involving anterior groin pain; positive impingement on physical maneuver involving flexion, adduction, and internal rotation of the hip; or imaging studies that suggest cam-type or pincer-type hip structural morphology) and had undergone a hip arthroscopy. Subjects were reviewed and included for analysis if they fulfilled the following inclusion criteria: (I) FAI injury, (II) no prior hip surgery or dysplasia, and (III) complete datasets. Subjects were excluded if they had history of previous hip surgery or hip dysplasia. A comparative group of non-football players was created with subjects matched on a 1:1 basis using age (± 2 years), sex, and body mass index (BMI) (± 2 kg/m^2^). Preoperative alpha angle and Wiberg angle were additionally included as covariates in the comparative analysis.

All patients underwent the same diagnosis protocol: clinical history and physical examination compatible with hip pathology. Complementary imaging studies were also performed including both plain radiographs and magnetic resonance imaging (MRI) to identify the crossover sign, the centre-edge angle and the α angle. Femoral head growth plate status was also evaluated to minimize the risk of physeal injury. All patients diagnosed with FAI went through a period of conservative treatment, including oral analgesics and physical therapy for strengthening the intrinsic periarticular hip muscles, stretching exercises, and anti-inflammatory techniques. In cases of persistent pain after 3 months, hip arthroscopy was indicated.

### Variables

Patient variables, injury characteristics, and preoperative and intraoperative outcomes were included in this analysis. Age, sex, weight, height, BMI, and sporting activity were registered at the time of the preoperative medical visit. Hip pain and function were assessed at that point through the Hip Outcome Score (HOS)—activities of daily living (HOS-ADL) and sports subscale (HOS-SS), Short-Form 36 (SF-36), Visual Analog Scale (VAS), modified Harris Hip Score (mHHS), International Hip Outcome Score (iHOT-33), Tegner Activity Scale, and the Hip Activity Scale (HSAS) questionnaires. All patient-reported outcome measures (PROs) were collected during the pre-operative evaluation period; consistent post-operative PRO data were not available for analysis.

Radiographic exams were used to assess preoperative Tönnis classification and pre and postoperative alpha angle, which measures the concavity of the femoral head-neck junction, and Wiberg angle, defined as the position of the femoral head in relation to the acetabulum [22, 23]. Injury morphology (CAM, CAM-chondromatosis, pincer, or mixed) was assessed through preoperative radiographic exams (Fig. 2). Acetabular labrum articular disruption (ALAD) classification is assessed preoperatively through MRI and later confirmed during the arthroscopic procedure [24]. Finally, surgical time was assessed at the time of the surgery and was defined as the time (minutes) between the opening of the first arthroscopic portal until the ending of the last portal suture.

### Surgical technique

All surgical procedures were performed by the same surgeon (RS). Patients were placed on a traction table in 15° of Trendelenburg. All patients received epidural anesthesia. The affected extremity was adducted and flexed 10° with the femur internally rotated. The contralateral leg was abducted 45° and externally rotated allowing the placement of the X-ray image intensifier between the legs. Hip distraction was applied using longitudinal traction of the affected limb. Surgical access was performed through an anterolateral portal located between the anterior superior iliac spine and the top of the great trochanter. Two additional working portals were opened anteriorly and distal to the first portal. Femoral osteoplasty and acetabular osteoplasty were performed in patients presenting cam and pincer morphology respectively. Labral repair or labrectomy were performed in case of labral injuries. Cartilage injuries were stabilized in cases with ALAD ≥ 2.

### Post-operative rehabilitation

After the surgery, all patients were instructed to discharge the affected limb for two weeks. Cryotherapy and passive mobilizations up to 90° of hip flexion were indicated to reduce pain and swelling. Pharmacologic prophylaxis for deep venous thrombosis was administered the first 10 days together with diclofenac to prevent heterotopic ossification. Two weeks after the surgery, patients progressed to partial weight bearing with crutches, active biking, and dynamic strength exercises. At 6 weeks after surgery, patients were allowed to perform unrestricted hip mobilizations and to progress in their weight-bearing and neuromuscular exercises as tolerated. Post-operative radiographic and clinical evaluations were routinely performed at approximately 10 weeks following surgery as part of standard follow-up.

Once range of motion and strength were satisfactory, patients in the football group started sport-specific readaptation. Unrestricted sport competitive activity was authorized approximately 3 months after surgery.

### Statistical analysis

Descriptive statistics were used for patient variables, injury characteristics, and preoperative and intraoperative outcomes. Central tendency measures were reported as the mean or median and dispersion measures were reported as standard error (SE) or range. A Shapiro–Wilk test for normality was first conducted. For quantitative variables with normal distribution, an independent samples t-test was used for a comparison across groups. For quantitative variables with non-normal distribution, a Mann–Whitney U test was conducted for a comparison across groups. Additionally, a paired samples t-test or Mann–Whitney U test was employed to compare informative angle values before and after the hip arthroscopy in both populations. For nominal and ordinal variables, a Chi-Square Test was used. The level of significance was set at α = 0.05 with a confidence interval (CI) of 95%. No a priori power analysis was conducted due to the retrospective nature and fixed sample size of the study. To provide context for the findings, a post-hoc power analysis was performed based on the observed difference in post-operative α and Wiberg angles. All statistical analyses were performed using the SPSS® Statistics software v.21 (SPSS Inc., Chicago, Illinois, USA). The authors obtained approval from the Institutional Review Board for this study.

## Results

**Fig. 1:**
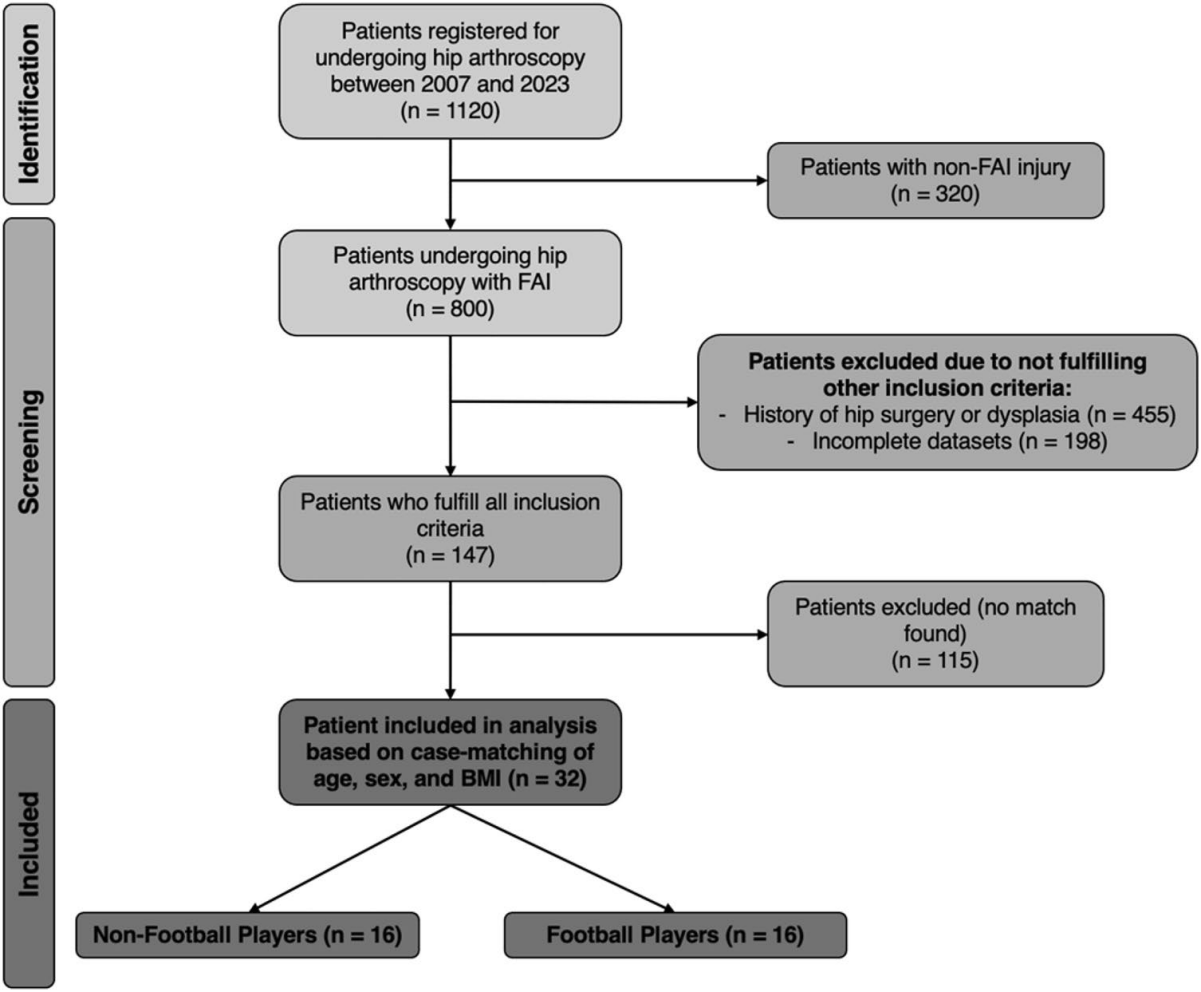
Patient selection process flowchart. Patient selection process flowchart outlining the inclusion and exclusion criteria for the study. A total of 800 patients were assessed, and 147 met inclusion criteria; 32 patients were ultimately included, with 16 in the football group and 16 in the non-football group

**Fig. 2:**
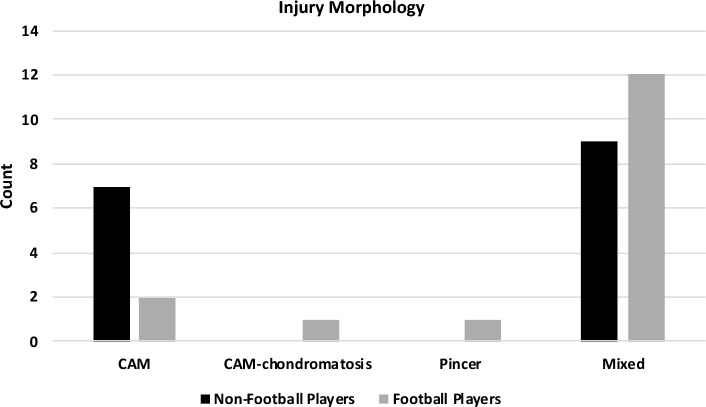
Injury morphology. Injury morphology classification based on radiographic evaluations. This figure shows the different types of femoroacetabular impingement (FAI): cam, pincer, and mixed morphology, as assessed pre-operatively

Between 2007 and 2023, 800 subjects with FAI underwent hip arthroscopy at our institution. From those, 147 (18.4%) further met all the inclusion criteria (Fig. 1), but only 16 were included in the football group because of case–control restrictions. A control group of 16 non-football players was created matched to the football group by age, sex, and BMI. Of the patients included for analysis, no significant differences were noted between the morphology of the FAI injury (p > 0.05, Fig. 2). Demographic characteristics of both groups are shown in Table 1.

**Table 1:** Patient demographics

Variable	Non-football players	Football players	p-value
Sex			0.673
Male	13	13	
Female	3	3	
Age (mean; range)	30.8 (23–39)	27.8 (19–42)	0.165
BMI (mean; range)	23.8 (21.1–27.1)	23.6 (20.3–25.8)	0.760

^**^Significant difference between football and non-football players (p < 0.05) using independent sample t-test (if age and BMI) or Fisher’s Exact test (sex)

### Hip pain

**Fig. 3:**
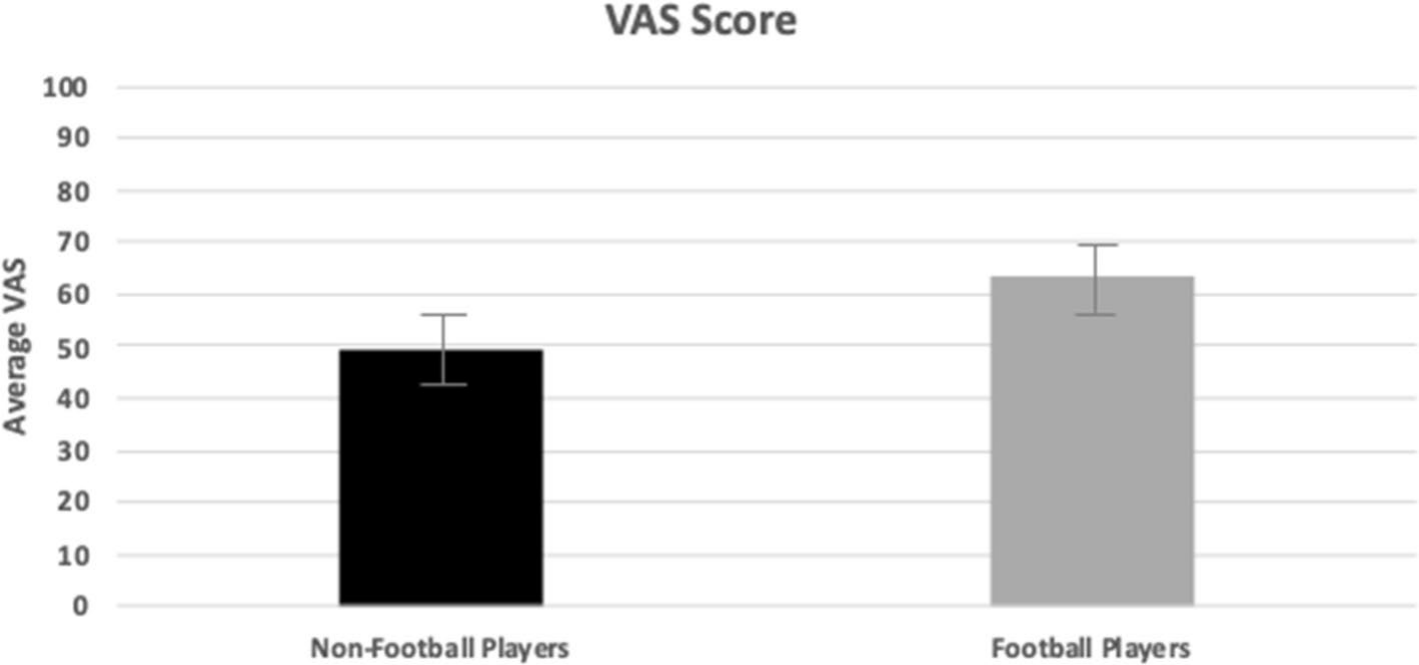
VAS Score (0–100). Visual Analog Scale (VAS) scores pre- and post-operatively for non-football and football players. This graph demonstrates the reduction in hip pain following hip arthroscopy in both groups, with no significant difference between groups (p > 0.05)

No significant differences in hip pain were observed between groups, with preoperative mean ± SE VAS scores of 49.38 ± 7.33 and 63.75 ± 6.36 for non-football players and football players respectively (*p* > 0.05) (Fig. 3).

### Hip function

**Fig. 4:**
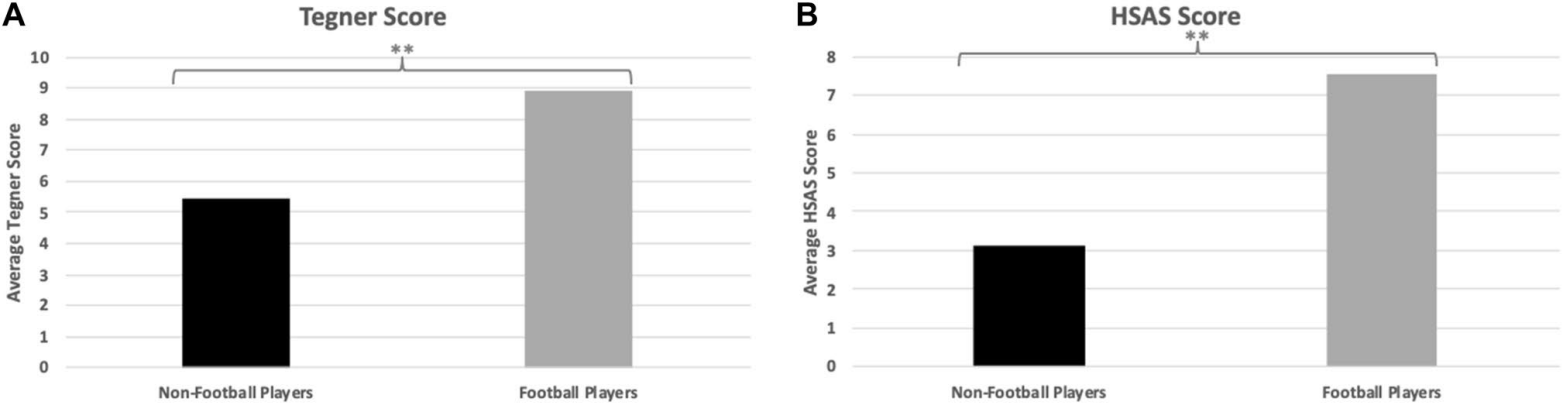
Pre-operative Nominal Variables. **a** Pre-operative Tegner Activity Scale (Tegner) scores for non-football and football players. The graph shows that football players have significantly higher pre-operative activity levels compared to non-football players (p < 0.001). **b** Pre-operative Hip Sports Activity Scale (HSAS) scores for non-football and football players. Football players have significantly higher scores compared to non-football players, reflecting a higher level of sports activity (p < 0.001). **Significant difference between non-football and football players (p < 0.001)

Significant differences between the non-football and football players were observed between groups for Tegner and HSAS score (*p* < 0.001). Preoperative mean ± SE Tegner score was 5.44 ± 0.60 (non-football) and 8.94 ± 0.28 (football) and HSAS score was 3.13 ± 0.68 (non-football) and 7.56 ± 0.44 (football) as shown in Fig. 4a, b. No other significant differences were observed between groups in any of the other functional scores (*p* > 0.05). Significant differences were noted across preoperative and postoperative in both the non-football and football groups for alpha (p < 0.001 & p < 0.001, respectively) and Wiberg (p < 0.002 & p < 0.001, respectively) angles. With 16 participants per group and an effect size of 0.32, the calculated power for the α angle was 14.2% (α = 0.05, two-tailed). Similarly, the with an effect size of 0.58, the calculated power for the Wiberg angle was 35.9% (α = 0.05, two-tailed). Mean, median, standard deviations, and frequencies are shown below along with the corresponding level of significance (Tables 2, 3, 4).

**Table 2:** Hip Function Outcome Scores, pre-operative variables

Variable	Non-football players	Football players	p-value
Surgical time	75.63 ± 2.37	71.25 ± 3.11	0.273
HOS ADL	83.82^††^	78.68^††^	0.722
HOS SS	51.56 ± 6.39	46.70 ± 6.48	0.597
SF-36 physical functioning	80.00^††^	67.50^††^	0.402
SF-36 physical health	50.00^††^	25.00^††^	0.479
SF-36 emotional problems	100.00^††^	100.00^††^	0.341
SF-36 energy/fatigue	52.50 ± 4.72	51.25 ± 5.01	0.857
SF-36 emotional well being	66.25 ± 4.07	65.25 ± 4.22	0.866
SF-36 social functioning	87.50^††^	75.00^††^	0.287
SF-36 general health	61.25 ± 6.32	62.50 ± 4.08	0.869
SF-36 pain	54.53 ± 6.56	45.78 ± 5.58	0.318
Physical Component Score	41.02 ± 2.63	37.99 ± 1.94	0.361
Mental Component Score	53.47^††^	51.84^††^	0.724
VAS	49.38 ± 7.33	63.75 ± 6.36	0.149
mHHS	71.71 ± 4.80	58.78 ± 5.31	0.081
iHOT	44.96 ± 4.76	45.41 ± 4.27	0.944
α angle (pre-op)	69.00 ± 2.30	68.06 ± 2.83	0.799
α angle (post-op)	47.63 ± 1.00	49.06 ± 1.25	0.724
α angle difference	21.38 ± 2.42	19.00 ± 2.61	0.510
Wiberg angle (pre-op)	35.00^††^	38.50^††^	0.113
Wiberg angle (post-op)	28.00^††^	28.00^††^	0.722
Wiberg angle difference	3.50^††^	8.00^††^	0.077
Tönnis^†^			0.828
0	11 (69%)	12 (75%)	
1	3 (19%)	3 (19%)	
2	2 (12%)	1 (6%)	
3	0 (0%)	0 (0%)	
ALAD^†^			0.394
0	1 (6%)	0 (0%)	
1	6 (38%)	11 (69%)	
2	2 (12%)	1 (7%)	
3	5 (32%)	2 (12%)	
4	2 (12%)	2 (12%)	

*PCS* physical component summary of SF-36*, MCS* mental component summary of SF-36

^†^Ordinal categorical variables instead of scalar variables

^††^Median instead of mean ± SE due to non-normal distribution

^**^Significant difference between non-football and football players (p < 0.05) using independent sample t-test (if normal distribution) or Mann–Whitney U test (if non-normal distribution) or Chi-Square test (if nominal or ordinal data)

**Table 3:** Hip Function Outcome Scores, pre-operative to post-operative changes in alpha angles

Variable	α Angle (in degrees)		p-value
	Pre-Op (Mean ± SE)	Post-Op (Mean ± SE)	
Non-football players	69.00 ± 2.30	47.63 ± 1.00	< 0.001^**^
Football players	68.06 ± 2.83	49.06 ± 1.25	< 0.001^**^

^**^Significant difference between pre-operation and post-operation (p < 0.05) using a paired t-test

**Table 4:** Hip Function Outcome Scores, pre-operative to post-operative changes in Wiberg angles

Variable	Wiberg Angle (in degrees)		p-value
	Pre-Op^†^	Post-Op^†^	
Non-football players	35.00 [26, 50]	28.00 [24, 38]	< 0.002^**^
Football players	38.50 [26, 54]	28.00 [24, 39]	< 0.001^**^

^†^Median [range] instead of mean due to non-normal distribution

^**^Significant difference between pre- operation and post- operation (p < 0.05) using Mann–Whitney U test

## Discussion

The principal finding of this study was that both football and non-football players with FAI have similar pre-operative outcome scores, and both groups achieved a significant improvement in α and Wiberg angles from hip arthroscopy. However, post-hoc power analyses revealed that the study was underpowered to detect small inter-group differences, requiring future studies with larger cohorts to validate these findings. Given that the Tegner and HSAS scores determine level of activity in patients suffering from FAI, it is within reason for values of football players (i.e. individuals of high activity) in those categories to be different from non-football players.

Overall, hip arthroscopy has shown to be a successful treatment in both athletes and non-athletes [25]. This study is among the first to compare football and non-players with FAI. Regarding α and Wiberg angles, Barrientos et al. concluded that hip pain in conjunction with an α angle above 57° and a Wiberg angle above 45° is strongly indicative of cam or pincer type impingement [10, 26]. These results showing significant improvement in both angle markers demonstrate a significant reduction in the hip impingement.

The visual analog scale has long been used to assess severity of pain in patients throughout many areas of the body [27]. Several studies have reported improvements in VAS score after hip arthroscopy in athletes [28–30]. In a study comparing female and male athletes, female athletes demonstrated a greater improvement than men in VAS score from preoperatively to latest follow-up [28]. Nonetheless, all athletes who underwent hip arthroscopy with capsular repair showed significantly greater improvements in VAS score [28, 29]. In addition, a study reported that hip arthroscopy can result in substantial improvement in the modified Harris hip score [31]. A similar study in football players under 16 years of age showed profound improvement in HOS ADL and HOS SS scores, among other hip function scales, between preoperative and one-year post-surgery [4]. Another study exploring outcomes after hip arthroscopy in patients with FAI reported significant improvements in the mHHS and iHOT scales both 12 and 24 months post-surgery, further demonstrating the validity in monitoring post-operative functionality via metrics used in this study [32]. Ultimately, these findings suggest the importance of longitudinal studies of patient-reported outcome scores in athletes and can be extended to non-athletes.

Rate and return to play are other key aspects to further investigate in all football players when intervention is performed. In a recent study, Barastegui et al. concluded 100% return to play in young athletes within the first year at a mean time of 5.93 months after hip arthroscopy [4, 28]. Translating this research to the general football population would be insightful information to help surgeons decide whether hip arthroscopy is worth it for football players. Furthermore, post-operative tracking of patient recovery would provide substantial evidence regarding the efficacy of hip arthroscopy.

This study has a few limitations. All non-football players were matched to a football player based on age, sex, and BMI. Overall, while this improves the reliability of the data analysis by assuring a homogenous group of individuals, it reduces the power of the study by limiting the sample size. From the dataset of 1120 patients, 147 subjects met the inclusion criteria; from the 147 subjects, the dataset was furthered narrowed to 32 subjects, based on case-matching for age, sex, and BMI. Furthermore, 3 of the 16 non-football players stated that they practice another sport at a high level. While the study was controlled for the fact that the patients were not football players, it is unknown if they practice another sport to certain degree. Lastly, the lack of post-operative PRO data prevented analysis of functional recovery beyond radiographic improvement.

Despite certain limitations, this study pioneers the field in analyzing pre-operative data in two actively-different groups of individuals. Additionally, all data was collected pre-operatively, intra-operatively, and post-operatively in a systematic manner by the same group of medical professionals further expanding the credibility and reliability of this study.

## Conclusions

Overall, there is very little difference in pre-operative and intra-operative scores across the two groups. However, there is significant differences noted in inter-group comparisons of Tegner and HSAS scores, as well as intra-group differences of pre- and post- Alpha and Wiberg angles. Additionally, hip arthroscopy is associated with significant radiographic improvements, specifically alpha and Wiberg angle, in both football and non-football players with FAI. However, further research is needed to evaluate long-term functional outcomes and return-to-sport rates to determine comprehensive treatment effectiveness and safety.

## Data Availability

No datasets were generated or analysed during the current study.
